# Increasing high-sensitive C-reactive protein level predicts peritonitis risk in chronic peritoneal dialysis patients

**DOI:** 10.1186/1471-2369-14-185

**Published:** 2013-09-04

**Authors:** Yu-Jen Su, Shang-Chih Liao, Ben-Chung Cheng, Jyh-Chang Hwang, Jin-Bor Chen

**Affiliations:** 1 Division of Nephrology, Kaohsiung Chang Gung Memorial Hospital and Chang Gung University College of Medicine, 123 Ta Pei Rd, Kaohsiung, Niao Song District, Taiwan; 2 Division of Nephrology, Chi-Mei Medical Center, 901 Zhonghua Rd., Tainan, Yongkang District 71010, Taiwan

**Keywords:** High-sensitivity C-reactive protein, Peritoneal dialysis, Peritonitis

## Abstract

**Background:**

The aim of this study was to evaluate whether a high baseline level of high-sensitivity C-reactive protein (hs-CRP) or changes in the level predicts the risk of peritonitis in patients on continuous ambulatory peritoneal dialysis (CAPD).

**Methods:**

A prospective, cross-sectional, case–control study was conducted in a single hospital-based PD unit. A total of 327 patients were included in the study. Serum hs-CRP was measured annually for 2 years. Patients were divided into 4 groups according to the changes in annual hs-CRP levels (at baseline and at 1 year intervals): group 1 (from <5 mg/L to <5 mg/L, n = 171), group 2 (from <5 mg/L to ≥5 mg/L, n = 45), group 3 (from ≥5 mg/L to <5 mg/L, n = 45), and group 4 (from ≥5 mg/L to ≥5 mg/L, n = 80). Demographics, biochemistry results, PD adequacy indices, and peritonitis risk were compared between the groups.

**Results:**

The initial serum albumin level was similar in the 4 groups (p = 0.12). There was a negative linear correlation between the serial albumin change (∆alb) and serial hs-CRP change (∆hs-CRP; r = −0.154, p = 0.005). The hazard ratio (HR) for peritonitis was significantly higher in group 2 (HR = 1, reference) than in group 4 (HR = 0.401, 95% CI 0.209 − 0.769). Group 2 had a greater serum albumin decline rate (∆alb: –3% ± 9%) and hs-CRP elevation rate (∆hs-CRP: 835% ± 1232%) compared to those for the other groups.

**Conclusions:**

A progressive increase in the hs-CRP level was associated with a corresponding decline in the serum albumin level. Progressive rather than persistently high levels of serum hs-CRP predicted peritonitis risk in CAPD patients.

## Background

Chronic inflammation remains an important adverse effect in patients undergoing dialysis. Several markers of inflammation have been used in clinical practice, including C-reactive protein (CRP), interleukin-6, and tumor necrosis factor. The CRP level is the most common indicator of inflammatory status. CRP is a nonglycated protein produced by human hepatocytes in response to infection, inflammation, or tissue damage. It is composed of 5 identical noncovalently linked subunits that form a symmetrical pentagonal structure with a molecular weight of 105 kDa. CRP is present at very low levels in the normal population. Levels < 1 mg/dL are considered insignificant, levels from 1 to 10 mg/dl are considered moderately elevated, and levels > 10 mg/dl are markedly elevated. Several methods are currently available commercially for use in clinical laboratories to measure CRP. The most commonly used are immunonephelometric and immunoturbidimetric assays. Recent studies have used newly developed high-sensitivity tests for CRP. These ultrasensitive enzyme-linked immunosorbent assays are capable of measuring CRP at a concentration of 0.007 mg/L [1].

An elevated CRP level, which is a marker of malnutrition, inflammation, and atherosclerosis (the MIA syndrome), is a risk factor for morbidity and mortality in the general population [2] and in dialysis patients [3,4]. Although a statistically significant association between the CRP level and cardiovascular disease has been observed in various studies [5-7], the relative contributions of CRP as a marker, causative agent, or consequence of the MIA syndrome are unclear in the dialysis population. In recent literature, a strong emphasis has been placed on an elevated CRP level as an important predictor of clinically apparent inflammatory infectious disorders based on cross-sectional analyses. However, the relationship between a single CRP measurement or variability in CRP levels and the risk of peritonitis in peritoneal dialysis (PD) patients remains uncertain. To the best of our knowledge, no studies have published data on the prognostic value of dynamic high-sensitivity CRP (hs-CRP) changes for peritonitis risk in PD patients.

Peritonitis is a serious infectious and a major complication that accounts for a significant risk of mortality in PD patients [8]. PD-related peritonitis, exit site infection, and other infections are the main causes of chronic inflammation. A single elevated CRP level combined with alterations in other acute-phase reactants is likely to be an epiphenomenon. It is unknown whether this epiphenomenon represents a chronic condition or whether it is an indicator of an ongoing acute process. The objective of the present study was to determine the prognostic value and clinical correlations of longitudinal hs-CRP variability in PD patients.

## Methods

### Patients and study design

A prospective, cross-sectional, case–control study design was used. This study included all prevalent PD patients from a PD institution at a medical center in southern Taiwan. All patients underwent monthly assessments of clinical and biochemical data from the start of the study in October 2008 until the censor date in October 2011, which was 2 years after the last patient was recruited into the study. The exclusion criteria were the presence of a known chronic inflammatory disease or major clinical events requiring hospital admission (for example, peritonitis, or other active infection) during the recruitment period from October 2008 to October 2009. To avoid interference with hs-CRP measurements, biochemical values that checked if events occurring in the 1 month before October 2008 were clinically active systemic, PD-associated infections, e.g. exit site infection or inflammatory process, were excluded. hs-CRP levels were measured in each patient at the time of recruitment and annually thereafter. All patients participating in the study were followed regularly for 2 years. A total of 327 patients were included in the final analysis. Patients were assigned to 4 groups according to the changes in hs-CRP levels between baseline and after 1 year. Group 1 was defined by a hs-CRP level change from <5 mg/L at baseline to <5 mg/L at 1 year (n = 171), group 2 from <5 mg/L to ≥5 mg/L (n = 45), group 3 from ≥5 mg/L to <5 mg/L (n = 45), group 4 from ≥5 mg/L to ≥5 mg/L (n = 80). The protocol for the present study was approved by the Committee on Human Research at Kaohsiung Chang Gung Memorial Hospital (CGMH-101-1595B), and all experiments were conducted in accordance with the Declaration of Helsinki. All participants provided informed consent for participating in the study.

### Laboratory measurement of hs-CRP

The hs-CRP level was assayed serially over the study period using the immunoturbidimetric method (Spectra East Laboratories, Rockleigh, NJ, USA). Serum hs-CRP <5 mg/L was considered the cutoff level in our laboratory.

### Statistical analyses

**Figure 1:**
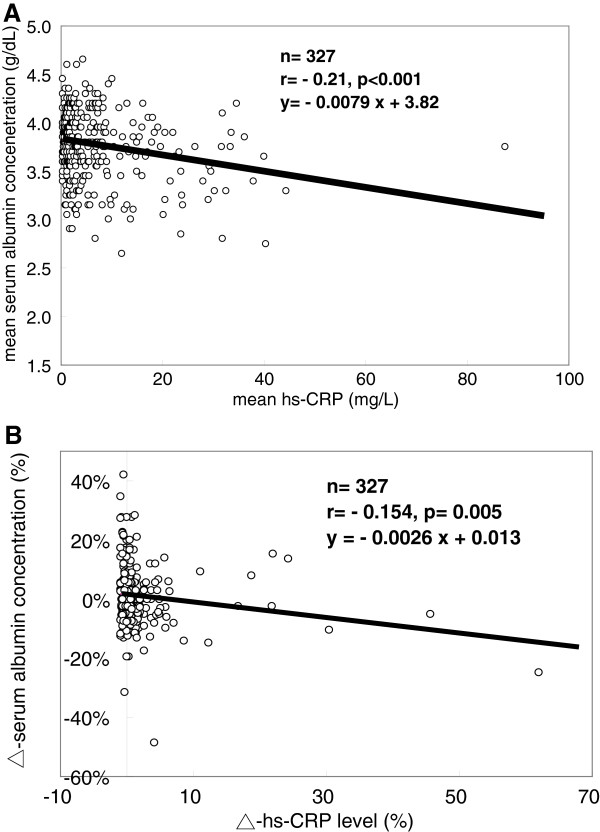
**Relationship between the albumin and hs-CRP levels.** In all patients as a group, there is a negative relationship between the mean serum albumin level and the mean hs-CRP level (r = –0.21, p < 0.001) **(A)**. There is also a negative linear correlation between ∆alb and ∆/hs-CRP (r = –0.154, p = 0.005) **(B)**.

The chi-square test and analysis of variance (ANOVA) with post hoc Bonferroni test were used for comparisons between categorical and continuous variables between groups, as shown in Table 1. Comparisons of hs-CRP levels among the 4 groups were performed using Kruskal–Wallis one-way ANOVA. Factors shown to be significant (p < 0.10) by simple logistic regression analysis, such as normalized protein catabolic rate (nPCR), body mass index (BMI), diabetes mellitus (DM), sex, blood urea nitrogen (BUN), phosphate, potassium, age at the initiation of the study, baseline albumin levels, and ∆albumin were reanalyzed using stepwise forward multiple logistic regression tests (Table 2). These were performed to evaluate the associations of characteristics of patients in group 2. Actual peritonitis-free rates in the 4 groups were determined using the Kaplan–Meier method, and log-rank tests were used to compare the cumulative peritonitis rates between groups. After adjusting for covariant factors, including DM, age during the study period, BMI, and the mean serum albumin level, Cox proportional hazard analysis was performed to determine the hazard ratios (HR) for peritonitis development in the 4 groups (Figure 1B). Except for the hs-CRP level (median, interquartile range), all data are expressed as mean ± standard deviation. A p value ≤ 0.05 was considered significant. Computations were performed using the SPSS 17.0 package for Windows (SPSS, Chicago, IL, USA).

**Table 1:** Baseline demographic characteristics of the 4 groups of patients

	**Group 1**	**Group 2**	**Group 3**	**Group 4**
	**n** = **171**	**n** = **45**	**n** = **31**	**n** = **80**
Demographic factors				
Mean age at the start of the study	48	51	49	55
(range)	(18–83)	(20–80)	(27–67)	(24–80)
Male, %	39	44	52	52
Diabetes, %	7.0	6.7	25.8^e^	22.8^e^
PD vintage, years	4.6 ± 3.2	5.5 ± 3.7	4.1 ± 2.9	5.3 ± 3.1
Kt/V urea (Gotch formula)	2.14 ± 0.46	2.11 ± 0.35	2.03 ± 0.39	2.02 ± 0.33
nPCR, g/day	1.11 ± 0.23^a^	1.08 ± 0.22	1.02 ± 0.23	1.00 ± 0.24
Body mass index, kg/m^2^	21.9 ± 3.5^b^	22.8 ± 3.9	24.0 ± 3.0	24.3 ± 3.8
Clinical co-morbidity, %				
Coronary artery disease	2	2	0	3
Congestive heart failure	6	7	3	9
Peripheral vascular disease	2	0	3	11
Stroke	2	4	10	9
Neoplasm	8	18	0	5
Chronic lung disease	5	2	6	8
Liver cirrhosis or hepatoma	3	0	10	4
No comorbidity, %	74	73	81	76
Recurrent peritonitis, %	5	9	0	6
Hospitalized stay, day	5 ± 11^a^	9 ± 14	8 ± 11	12 ± 21
Laboratory data				
Baseline albumin, g/dL	3.80 ± 0.38	3.79 ± 0.39	3.68 ± 0.50	3.68 ± 0.42
Final albumin, g/dL	3.83 ± 0.35^a,c^	3.66 ± 0.39	3.87 ± 0.40	3.67 ± 0.43
∆ albumin, %	1% ± 7%^c^	−3% ± 9%	6% ± 12% ^a,c^	0% ± 11%
Baseline hs-CRP, mg/L,	1.49	2.10 ^f^	9.75 ^g^	11.6^g^
(Q1–Q3)	(0.84 to 2.25)	(1.30 to 3.61)	(7.30 to 16.1)	(7.85 to17.8)
Final hs-CRP, mg/L,	1.45	9.20 ^g^	2.49^f^	12.8^g^
(Q1–Q3)	(0.74 to 2.39)	(7.29 to 12.1)	(1.36 to 3.71)	(7.89 to 25.1)
∆ hs-CRP, %	−6^h^	416	−80^h^	0^h^
(Q1–Q3)	(−38 to 49)	(1.78 to 7.02)	(−91 to −59)	(−29 to 69)
Hematocrit, %	32.2 ± 4.3^c^	29.7 ± 4.6	30.9 ± 3.9	30.7 ± 4.0
Serum cholesterol, mg/dl	185 ± 39	177 ± 42	196 ± 59	184 ± 39
Serum triglyceride, mg/dL	145 ± 83	138 ± 93	158 ± 109	174 ± 93
Serum K, mEq/L	4.1 ± 0.7^b^	4.0 ± 0.6	3.9 ± 0.8	3.7 ± 0.6
BUN, mg/dL	66 ± 17	72 ± 19	62 ± 16	64 ± 15^c^
Serum Cr, mg/dL	12.1 ± 2.8	11.8 ± 3.0	11.6 ± 3.5	11.2 ± 2.6
Serum phosphorus, mg/dL	5.2 ± 1.1	5.6 ± 1.5	5.4 ± 1.5	5.0 ± 1.2^c^
Serum calcium, mg/dL	9.3 ± 0.8	9.3 ± 0.8	9.1 ± 0.9	9.4 ± 0.8

Abbreviations: *PD* peritoneal dialysis, *nPCR* normalized protein catabolism rate, *BUN* blood urea nitrogen, *Cr* creatinine.

Statistics:

^a^p < 0.05, ^b^p < 0.001 vs. group 4 (ANOVA with Bonferroni tests), ^c^p < 0.05, vs. group 2 (ANOVA with Bonferroni tests), ^e^p < 0.05 vs. group 2 (Chi square tests), ^f^p < 0.05, ^g^p < 0.001 vs. group 1 (Kruskal–Wallis one-way analysis of variance), ^h^p < 0.001 vs. group 2 (Kruskal–Wallis one-way analysis of variance).

**Table 2:** Logistic regression analysis of group 2

	**Simple**				Multiple*			
		**95% CI**				**95% CI**		
	**OR**	**Lower**	**Upper**	**Sig.**	**OR**	**Lower**	**Upper**	**Sig.**
nPCR, g/day	1.172	0.313	4.381	0.814				
BMI, kg/m^2^	1.001	0.92	1.089	0.989				
BUN, mg/dL	1.025	1.007	1.043	0.007	1.019	1.000	1.039	0.055
Phosphate, mg/dL	1.35	1.051	1.733	0.019	1.232	0.948	1.601	0.119
Potassium, mmol/L	0.969	0.617	1.522	0.891				
Hematocrit, %	0.907	0.845	0.974	0.007	0.92	0.855	0.99	0.026
age at study, year	1.009	0.985	1.034	0.471				
Diabetes mellitus (=1)	0.459	0.135	1.554	0.211				
Gender, (male gender = 1)	0.981	0.521	1.848	0.953				
baseline albumin, g/dL	1.281	0.581	2.823	0.54				
∆albumin, %	0.945	0.911	0.981	0.003	0.947	0.912	0.982	0.004

Abbreviations: *nPCR* normalized protein catabolic rate, *BMI* body mass index, *BUN* blood urea nitrogen, ∆albumin, ratio of albumin concentration change, * adjusted for: *BUN* phosphate, hematocrit, and ∆albumin.

## Results

### Demographic characteristics of the 4 groups of patients

As shown in Table 1, group 2 had a significantly lower prevalence of DM compared to that in groups 3 and 4. Patients in group 4 had a higher mean BMI and length of hospital stay but lower levels of nPCR compared to those for patients in group 1. They also had significantly lower levels of final serum albumin and serum potassium. Baseline hs-CRP levels were significantly lower in groups 1 and 2 compared to the levels in groups 3 and 4. Group 4 patients had higher baseline and final hs-CRP levels compared to those in the other 3 groups. Group 2 had significantly lower final serum albumin levels, and higher final hs-CRP levels compared to the levels in group 1. Although group 4 had the highest final hs-CRP levels, the increment of hs-CRP (∆hs-CRP) was the highest in group 2. Group 1 had higher baseline and final serum albumin levels but lower final hs-CRP levels and ∆hs-CRP compared to those in group 2, although the initial serum albumin levels were similar (p = 0.12).

### Relationship between the changes in serum albumin (∆alb) and hs-CRP (∆hs-CRP) levels

In all patients, the mean serum albumin level was negatively correlated with the mean hs-CRP level (r = −0.21, p < 0.001). Furthermore, there was a negative linear correlation between ∆alb and ∆hs-CRP (r = −0. 154, p = 0.005; Figure 1A and B).

### Cox proportional hazard analysis for the evaluation of the covariant factors related to peritonitis development

**Figure 2:**
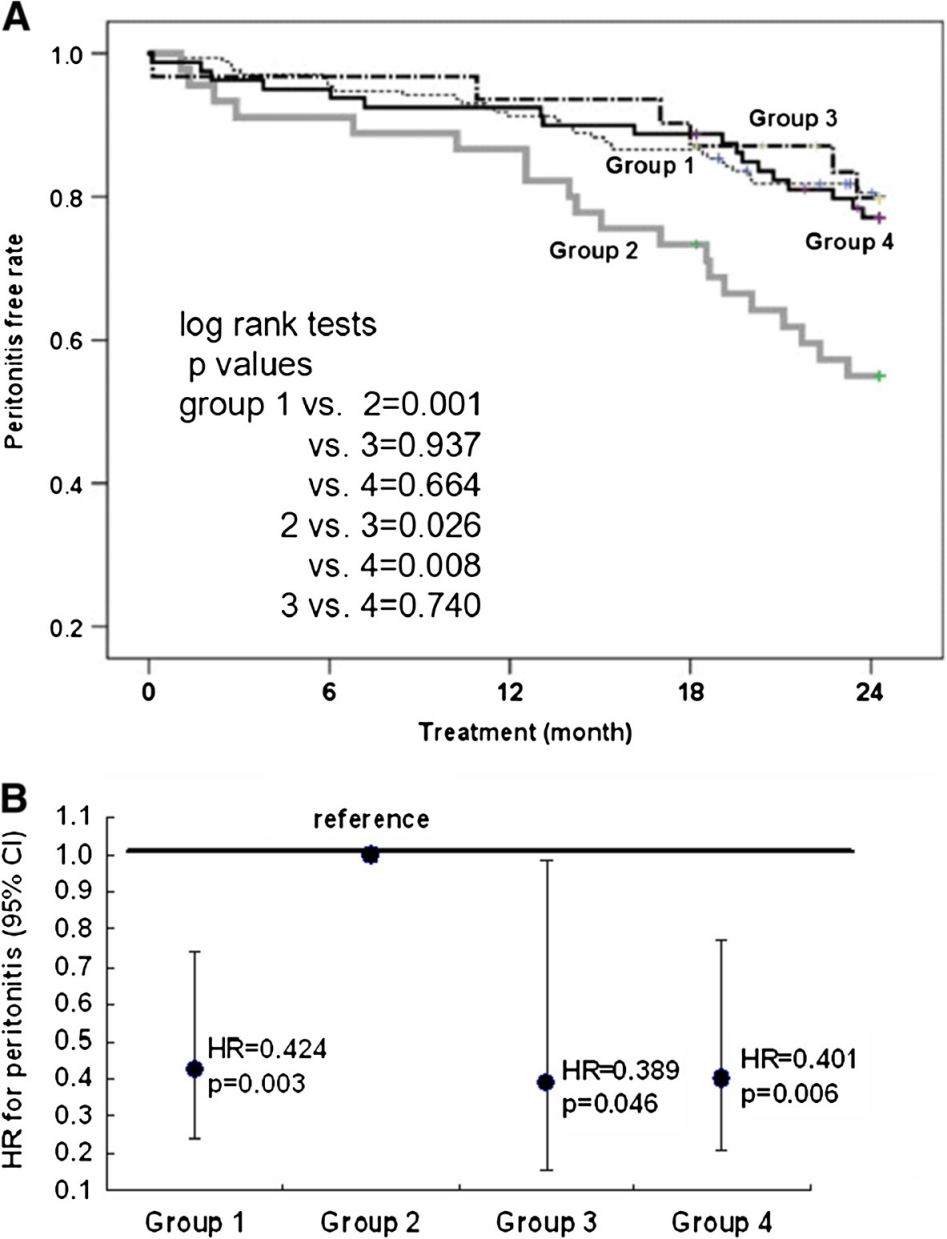
**Peritonitis-free rates between the different groups.** The peritonitis-survival free rate is significantly lower in group 2 compared to that in the other 3 groups **(A)**. After adjusting for covariant factors, including DM, age at the time of the study, BMI, and the mean serum albumin level, the HR for peritonitis is significantly higher in group 2 (reference group, HR = 1) compared to that in group 4 (HR = 0.401, 95% CI 0.209–0.769) and in the other 2 groups **(B)**.

Log-rank tests showed no significant difference in the peritonitis-free rates between the different groups, except in group 2. The peritonitis-free rate in group 2 was significantly lower than that in the other 3 groups (Figure 2). After adjusting for covariant factors, including DM, age at the time of the study, BMI, and the mean serum albumin level (Figure 2B), the HR for peritonitis development was significantly higher in group 2 (HR = 1) than in group 4 (HR = 0.401, 95% CI 0.209 − 0.769) and the other 2 groups.

### Characteristics of patients in group 2

The patients in group 2 were characterized by higher BUN levels (OR 1.019; 95% CI 1.00 − 1.039) but lower hematocrit (OR 0.92, 95% CI 0.855 − 0.99) and lower serum albumin levels (OR 0.947, 95% CI 0.912 − 0.982) compared to the levels in the other 3 groups Table 2. The patients in group 2 also showed the highest serum albumin decline rate (∆alb: –3 ± 9%) and hs-CRP elevation rate (∆hs-CRP: 835 ± 1232%).

## Discussion

The main finding of this study was that a progressive increase in the hs-CRP level was associated with a corresponding decline in the serum albumin level. Progressive rather than a persistent high serum hs-CRP level predicted peritonitis risk in continuous ambulatory peritoneal dialysis (CAPD) patients. Surprisingly, patients with sustained high levels of hs-CRP had a similar risk of peritonitis compared to that in patients with constant normal hs-CRP values.

Approximately 30% of hemodialysis patients [9] and up to 58% of PD patients [10] have high CRP levels. CRP is a predictor of subsequent cardiovascular disease, cardiovascular mortality, and all-cause mortality in the general population [11] and in dialysis patients [5,12]. Most of these published studies consider the rise in CRP combined with alterations in other acute-phase reactants as an epiphenomenon. Regardless of its precise role, the serum CRP level is a well-known indicator of inflammation, and chronic inflammation remains an important adverse event in patients undergoing dialysis. For patients on PD, PD-related peritonitis, exit-site infection, and other infections are the main causes of chronic inflammation [13]. Over the past decade, the relationship between inflammation markers (e.g. an elevated CRP level), malnutrition (e.g. hypoalbuminemia), and mortality has received much attention. However, not all studies have been sufficiently powered to assess this relationship. Moreover, an association between elevated CRP levels and the risk of peritonitis has not been investigated critically in PD patients. Our study is the first report to address the association between dynamic changes of hs-CRP and peritonitis risk in CAPD patients.

Evidence that inflammation is responsible for regulating serum albumin levels in dialysis patients was provided by several cross-sectional studies [9,14,15]. An acute-phase response, measured by CRP or serum amyloid A levels, was significantly correlated with serum albumin levels in PD patients [16]. Because variations in serum albumin levels are small, measurement of changes in CRP levels may provide greater insight into the dynamics of nutritional status and clinically relevant insults such as peritonitis. The main finding of our study was that an increase in the hs-CRP level was associated with a decrease in the serum albumin level. Furthermore, the levels of nPCR were higher in subjects with persistently low hs-CRP levels than in those with persistently high hs-CRP levels. These results reflected the impact of inflammatory status on the nutritional status of the PD patients in this study.

Current hypotheses state that the increased CRP observed in dialysis patients reflects the severity of an “inflammatory state,” caused by dialysis itself [17], vessel inflammation due to atherosclerosis [5,18], and oxidative stress generated by uremia [19]. Levels of inflammatory and oxidative stress biomarkers are increased further in hypoalbuminemic compared with normoalbuminemic dialysis patients. Hypoalbuminemia, acute-phase inflammation, and oxidative stress may act synergistically to increase cardiovascular morbidity and mortality risk in hemodialysis patients [20]. However, little has been reported on oxidative stress and antioxidant capacity in relation to serum albumin in PD patients. In contrast, Kim et al. [21] showed that oxidative stress was reduced in hypoalbuminemic PD patients. Therefore, a well-designed study is necessary to explore the relationship between oxidative stress and nutritional indices in PD patients.

There is some evidence that CRP itself may have a proinflammatory effect. For instance, CRP is able to activate the complement system and is a valuable marker rather than a contributor to the atherosclerotic process [22]. Furthermore, CRP is most helpful as a clinical parameter among various proinflammatory markers for monitoring microinflammation in patients with peritonitis [23]. The additional presence of systemic inflammation (dynamic increase in the hs-CRP level group), irrespective of its origin, may be the factor “tipping the scales” toward a catastrophic peritonitis event, possibly by directly activating mesothelium cells, which is the first line of defense against foreign particles and chemicals in the peritoneal cavity. Increased synthesis of proinflammatory cytokines and matrix proteins is observed after the activation of infiltrating and resident peritoneal cells, leading to morphological changes such as reduplication of the basement membrane, induction of epithelial-to-mesenchymal transdifferentiation, a breakdown of the basement membrane, and their migration into the submesothelium [24]. A loss of the protective mesothelium allows PD fluid and toxins released by bacteria to induce the activation of peritoneal fibroblasts and hyalinization of blood vessels, which invariably increases the peritonitis risk [25].

In the present study, persistently high serum hs-CRP levels (group 4) were not associated with a higher peritonitis risk, and the underlying mechanisms remained unclear. PD patients are exposed to persistent low-grade inflammation, and levels of serum inflammatory markers vary substantially over time under the influence of multiple processes such as transient infection, comorbidities, and the intermittent stimulus of dialysis. The differences in the condition of individual PD patients resulted in inter-individual variability in serum hs-CRP levels. The cardiovascular co-morbidity rate in group 4 was relatively high compared to that in the other 3 groups. This factor might result in sustained high hs-CRP levels, but may not be associated with an increased peritonitis risk in the following year. The results of the present study further enforced the importance of serial hs-CRP measurements.

Although chronic kidney disease should be considered in association with the inflammatory status, the extent of inflammation was not consistent. Therefore, a single measurement of inflammatory markers such as hs-CRP is not suitable to assess the true condition of patients. Regular monitoring of inflammatory markers is more reasonable for accurate clinical prediction. Snaedal et al. [26] used median or mean CRP levels measured weekly over a 3-month period to assess 224 patients who were undergoing hemodialysis. The results showed that an elevation of 1 mg/L in median or mean CRP levels was correlated with a 1.2–1.3% increase in the HR for all-cause mortality. Other studies based on consecutive measurement of CRP levels in dialysis patients showed that those with persistently elevated CRP levels had worse all-cause mortality than those with persistently low CRP levels or CRP elevations and/or decreases [27-29]. In the present study, the PD-related peritonitis-free period was lower in patients with progressive hs-CRP elevation than in the other patients, which is in agreement with the results of previous studies showing the association between all-cause mortality and changes in the CRP level. Because the CRP measurements in the present study were not performed during an active infectious period, our findings imply that a temporal or sub-clinical inflammatory reaction was ongoing when the measurement was taken. Therefore, it may provide an early warning sign to clinical physicians to closely observe ominous infectious situations such as PD-related peritonitis.

To the best of our knowledge, this is the first report describing a stratified fluctuation of hs-CRP levels related to PD-peritonitis risk. However, our study had some limitations. First, because hs-CRP measurements were performed annually for 2 years, it is possible that subtle inflammatory reactions occurring during the study period were not detected. Second, the sample size was relative small. Third, this was a cross-sectional prospective study, and selection bias might preclude other morbid patients.

## Conclusions

In conclusion, our data demonstrated an association between a progressive increase in hs-CRP levels and peritonitis risk in CAPD patients. The serum albumin level was negatively correlated with the hs-CRP level in these patients, implying that the extent of the decline in the serum albumin level reflects the progress of the sub-clinical inflammation and/or infection. This may explain the association between the progressive elevation of hs-CRP levels and the development of peritonitis in CAPD patients. Our results showed that persistent high levels of hs-CRP were not related to peritonitis risk. Although we suspect that adaptation and/or accommodation to mild sub-clinical inflammation may be a contributing factor, the underlying mechanism remains to be elucidated.

## Competing interests

The authors declare that they have no competing interests.

## Authors’ contributions

J-BC collected and interpreted the data, critically reviewed the manuscript, and drafted and included revisions in the manuscript. Y-JS and S-CL assisted with the collection of the data, interpreted the data, and were involved in the drafting of the manuscript. J-CH and J-BC participated in the acquisition and interpretation of data, revised the manuscript critically, and performed the statistical analyses. B-CC, J-CH, and J-BC conceived, designed, and coordinated the study, and commented on the final draft. All authors have read and approved the final manuscript.

## Pre-publication history

The pre-publication history for this paper can be accessed here:

http://www.biomedcentral.com/1471-2369/14/185/prepub
